# Heterogeneous Ti_3_C_2_T_*x*_ MXene-MWCNT@MoS_2_ Film for Enhanced Long-Term Electromagnetic Interference Shielding in the Moisture Environment

**DOI:** 10.1021/acsami.3c08279

**Published:** 2023-10-16

**Authors:** Sarab Ahmed, Baosong Li, Shaohong Luo, Kin Liao

**Affiliations:** † Department of Aerospace Engineering, Khalifa University of Science and Technology, 127788 Abu Dhabi, UAE; ‡ Department of Biomedical Engineering, Khalifa University of Science and Technology, 127788 Abu Dhabi, UAE; § Department of Mechanical Engineering, Khalifa University of Science and Technology, 127788 Abu Dhabi, UAE

**Keywords:** EMI shielding, heterogeneous film, Ti_3_C_2_T_*x*_ MXene, MWCNT@MoS_2_, environmental stability

## Abstract

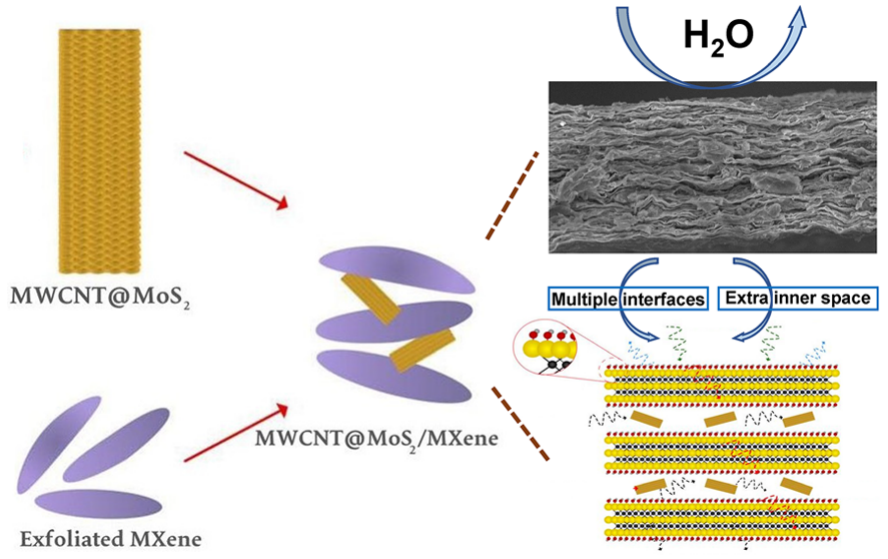

MXene, as a novel two-dimensional (2D) material, has unique inherent features such as lightweight, flexibility, high electrical conductivity, customizable surface chemistry, and facile solution processability. However, utilizing MXene (Ti_3_C_2_T_*x*_) films for long-term electromagnetic interference (EMI) shielding poses challenges, as they are susceptible to chemical deterioration through oxidation into TiO_2_. In this work, an ultrathin heterogeneous film of Ti_3_C_2_T_*x*_ MXene integrated with multiwalled carbon nanotubes supporting MoS_2_ clusters (MXene/MWCNT@MoS_2_) was developed. The heterogeneous film with 15 wt % of MWCNT@MoS_2_ clusters exhibited improved EMI shielding performance such as the highest EMI shielding effectiveness of 50 dB and the specific shielding effectiveness of 20,355 dB cm^2^ g ^–1^, mainly attributed to the excellent electrical conductivity, distinctive porous structure, and multiple interfacial interactions. The heterogeneous films underwent extended exposure to a moisture environment (35 days), and their structural stability and EMI shielding performance were enhanced by the integration of MWCNT@MoS_2_ clusters. As a result, the engineered heterostructure of multilayered hybrid films holds promise as a viable option for improving the EMI shielding effectiveness and stability of Ti_3_C_2_T_*x*_ MXene.

## 1 Introduction

Electromagnetic interference (EMI) shielding refers to the prevention of electromagnetic waves (EMWs) from penetrating a material. It is significant in the contemporary digitalized world, where electronic devices can be protected from malfunctioning by EMI shielding materials.^1^ Due to the increasing cases of electronic pollution such as EMI, radiofrequency interference (RFI), and electronic noise, extensive research has been carried out to identify shielding materials for countering these effects.^2^ Interference caused by EMW has raised notable concerns, especially in domains involving health-related devices, industrial pollution, and military equipment. To mitigate potential detrimental outcomes due to EMI, certain organizations followed standard electromagnetic compatibility (EMC) regulations, which specify the capability of equipment or the material in shielding off EMI.^3^ Electromagnetic shielding has evolved as a technique involving the envelopment of susceptible materials that emit excessive emissions into the surroundings. Metallic materials were used as covers to reduce the EM emission into the external environment. However, their practicality is undermined by their weight, poor corrosion resistance, and high cost.^4^ Research has therefore been carried out to explore alternative materials beyond metals that can fulfill a similar function.^5^ A core/shell heterostructure comprising carbon nanotubes (CNTs) and MoS_2_, supported on reduced graphene oxide (rGO), exhibited an impressive EMI shielding effectiveness (EMI SE) of 40 dB at a thickness of ∼1 mm.^6^ The CNT/MoS_2_ core/shell structure functions as a good source of interfacial polarization, whereas the rGO forms a conductive network, rendering the hybrid highly conducive to electron transport. Recently, it has been shown that 2D materials boasting exceptional attributes—such as lightweight nature, high electrical conductivity, and flexibility—can be used to shield off EMW.^7^ The application of 2D materials as EMI shielding materials is still in its infancy, presenting opportunities for further exploration of their applications.

MXenes (M_*n*+1_X_*n*_T_*x*_) are 2D inorganic compounds that consist of a few atoms in layers of carbides, nitrides, and carbonitrides.^8−10^ Owing to their high electrical conductivity, outstanding mechanical properties, and diverse surface chemistry,^11−13^ MXenes, particularly Ti_3_C_2_T_*x*_ and its composites, have garnered considerable attention as a compelling candidate for EMI shielding.^14,15^ For instance, Yury reported a Ti_3_C_2_T_*x*_ MXene-based film with a remarkable EMI SE of 92 dB at a thickness of 45 μm owing to the superb electrical conductivity of Ti_3_C_2_T_*x*_ films (4600 S cm ^–1^).^14^ In addition, Ti_3_C_2_T_*x*_/cellulose nanofibrils (CNFs)/MWCNT EMI composite films exhibited enhanced SE fueled by the yarn-ball-shaped microspheres which shield more EMWs by repeated reflections in the inner space.^16^ Although MXene-based materials present high EMI SE, their poor structural stability caused by the oxidation of Ti_3_C_2_T_*x*_ nanoflakes inevitably leads to the decrease of the electrical conductivity and the loss of EMI shielding capability.^17^ First-principles molecular dynamics simulations were applied to probe the water/Ti_3_C_2_O_2_-MXene interfacial chemistry, verifying that water molecules can induce the reconstruction of MXene structure by breaking the Ti–C bonds and facilitating the formation of the Ti–OH bonds on the surface of Ti_3_C_2_O_2_.^18^ Therefore, strategies based on preventing interactions of water molecules and Ti atoms are imperative for enhancing the stability of the MXene structure. To this end, Wu et al. prepared sodium ascorbate–capped Ti_3_C_2_T_*x*_ MXene, which exhibits remarkable resistance to oxidation when exposed to ambient air.^19^ Li et al. proposed a heterogeneous film constructed from Ti_3_C_2_T_*x*_ MXene and porous double-layered carbon nanosheets.^20^ With the protection of carbon nanosheets, the Ti_3_C_2_T_*x*_ phase and EMI shielding performance were well sustained, even after several months.

In this study, we prepared a heterogeneous film by embedding MWCNTs-supported MoS_2_ clusters into Ti_3_C_2_T_*x*_ MXene layers (donated as MXene/MWCNT@MoS_2_) via a simple vacuum-assisted filtration process. The MWCNT@MoS_2_ clusters in the heterogeneous film can expand the layer spacing of Ti_3_C_2_T_*x*_ MXene nanosheets and restrain restacking of the layered MXene, which leads to a higher EMI SE for the heterogeneous film. Furthermore, the introduction of MWCNT@MoS_2_ clusters plays a pivotal role in enhancing the structural stability of the heterogeneous film, rendering it more resilient to challenging environmental conditions. Benefiting from the merits conferred by MWCNTs@MoS_2_ clusters, the heterogeneous film is applicable to the EMI shielding with high SE and improved stability in a moisture environment.

## 2 Experimental Section

### 2.1 Chemicals and Reagents

Hydrophilic acid (HCl, 36 %), thiourea, sodium molybdate dihydrate (Na_2_MoO_4_·2H_2_O), and lithium fluoride (LiF, 99 %) were purchased from Sigma-Aldrich Co., Ltd. Polypropylene membrane (Celgard, 3501) was bought from Celgard LLC. MAX (Ti_3_AlC_2_, ≤40 μm) was purchased from Carbon-Ukraine Ltd. d-Glucose anhydrous was purchased from Fisher Scientific, and MWCNTs were purchased from Timesnano.

### 2.2 Preparation of MWCNT@MoS_2_ Clusters

Acid-treated MWCNTs (30 mg) were disseminated in glucose solution (40 mL, 0.05 M) by ultrasonication for 30 min. The solution was then supplemented with sodium molybdate dihydrate (0.4 g) and thiourea (0.8 g). After stirring for 10 min, the solution was put into a Teflon-lined stainless-steel autoclave and incubated in an electric oven at 200 °C for 24 h. Subsequently, the black precipitate was centrifuged, thoroughly rinsed with ethanol four times, and then dried at 80 °C for 12 h.^21^

### 2.3 Preparation of Ti_3_C_2_T_*x*_ MXene Nanosheets

Ti_3_C_2_T_*x*_ was prepared by selectively etching Al layers from the MAX phase (Ti_3_AlC_2_). Typically, 3.2 g of lithium fluoride (LiF) was dissolved in 40 mL of 9 M HCl in a plastic bottle, and then 2 g of Ti_3_AlC_2_ powders was slowly added into the above solution under stirring. The bottle was continuously stirred at 35 °C for 24 h. After that, the mixture was washed with deionized water via centrifugation at 3500 rpm for several cycles until the pH of the supernatant was above 6 (this step was repeated four or five times until the colloid MXene solution was stable). Once the solution is stable, it will be centrifuged for 1 h to remove excess salt. After collecting the dark solution, it will be centrifuged at 3500 rpm for 10 min to make sure that no multilayer powder remains.^22^

### 2.4 Preparation of Flexible MXene/MWCNT@MoS_2_ Heterogeneous Films

MXene/MWCNT@MoS_2_ heterogeneous films were fabricated via a vacuum-assisted filtration process. An appropriate amount of the MWCNT@MoS_2_ was dispersed in deionized water by sonicating for 30 min to obtain uniform dispersion. Then, the MXene solution was added and stirred vigorously for 2 h. Finally, the mixture was filtrated through a polypropylene membrane (Celgard, 3501) to obtain MXene/MWCNT@MoS_2_-*X* (*X* is the proportion of MWCNT@MoS_2_ in the heterogeneous film), here referring to MXene/MWCNT@MoS_2_-5%, MXene/MWCNT@MoS_2_-15%, and MXene/MWCNT@MoS_2_-25%.

### 2.5 Stability Evaluation of the Films under Different Conditions

The moisture experiment was conducted by keeping all samples in containers with different moisture environments, for up to 35 days. The types of containers are involved here (Figure S1): (1) a desiccator with desiccant (<10% RH), (2) a container in open air (55% RH), and (3) an environmental chamber made of a sealed small container with water inside (90% RH). The humidity was measured by hygrometers inside the containers at room temperature. All three containers have 3 pieces of each film sample, and the samples were stored for the specific time and taken out at intervals to measure EMI SE.

### 2.6 Material Characterization

Field emission scanning electron microscopy (FESEM) (JEOL, JSM 7610F) and transmission electron microscopy (TEM) (TECNAI G220 U-Twin instrument) were used to analyze the morphology and structure of the samples. X-ray diffraction (XRD) for the samples was conducted by a powder X-ray diffractometer (Bruker D2 PHASER, Cu Kα radiation, *k* = 1.54184 Å). The Raman spectrum of the samples was measured with a Raman spectrometer (Horiba LabRAM HR Evolution). The thicknesses of the samples were estimated by an atomic force microscope (Asylum MFP-3D). Electrical conductivity was measured using an Ossila Four-Point Probe System (MCP-T610 model, Mitsubishi Chemical, Japan). A dynamic mechanical analyzer was used to test mechanical properties (INSTRON-5948) of the samples (0.3 cm of width and 1.5 cm of length, repeated 3 times for all samples). The density of the sample is calculated by the following equation:
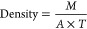
1

where *M*, *A*, and *T* are the mass, surface area, and thickness of the film, respectively.

The EMI shielding measurements were carried out using a pair of waveguides and a network analyzer (Agilent, E5071CENA). A pair of coaxial cables was connected between the rectangular waveguide adopters and the Agilent network analyzer model. A fabricated sample was inserted into the rectangular sample holder for taking the measurement, within a frequency range of f 8.2 – 12.4 GHz (X band). To prevent leakage channels from the margins, all the film samples were trimmed into rectangular shape (25 × 12 mm^2^) that were only a slightly larger than the sample holder. All of the EMI shielding measurements of the samples were carried out three times.

EMW can be absorbed, reflected, or attenuated within a material. Each of the aforementioned mechanisms contributes to the total EMI SE (SE_T_), which is defined as the sum of the SE of absorption (SE_*A*_), reflection (SE_*R*_), and multiple reflections (SE_*M*_), as shown in eq 1.
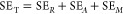
2However, when SE_T_ is greater than 15 dB, SE_*M*_ is negligible.^23^ Therefore, the following equations were used to determine SE_T_, SE_*R*_, and SE_*A*_:
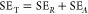
3
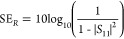
4
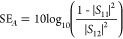
5where *S*_11_ and *S*_12_ are scattering parameters measured by the network analyzer. *S*_11_ indicates that the EMI radiation is transmitted by port 1 and received by port 1; *S*_12_ indicates that the EMI radiation is transmitted by port 2 and received by port 1.

Considering the density and thickness, the absolute effectiveness (SSE/*t*) can be evaluated using the following equation:
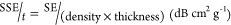
6The experimentally accessed scattering parameters *S*_11_ and *S*_12_ were used to evaluate the relative complex permittivity and permeability.

## 3 Results and Discussion

**Figure 1:**
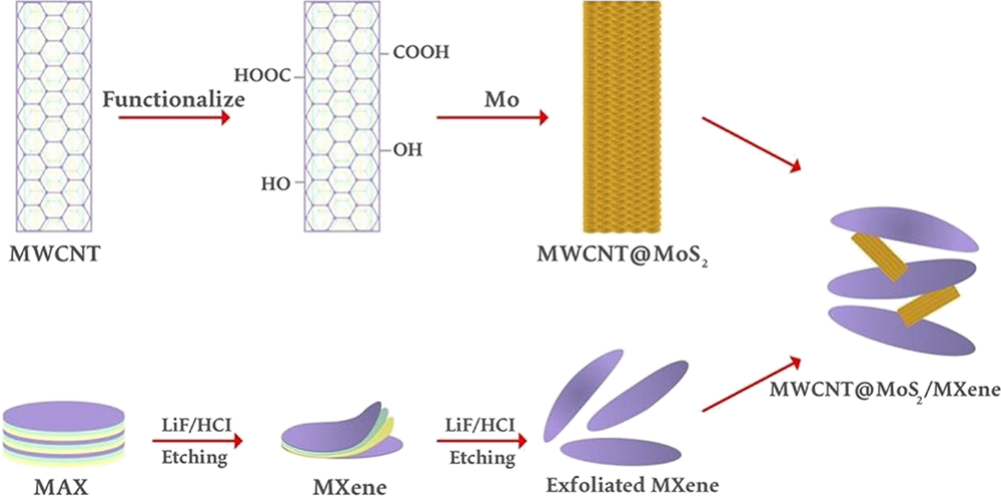
Schematic illustration of the preparation of the MXene/MWCNT@MoS_2_ film.

The synthesis process of the MXene/MWCNT@MoS_2_ films is summarized in Figure 1. The entire process encompasses the fabrication of MWCNT@MoS_2_, the synthesis of Ti_3_C_2_T_*x*_ MXene, and the assembly of MXene and MWCNT@MoS_2_. MWCNT@MoS_2_ clusters were synthesized via a hydrothermal reaction using functionalized MWCNTs as the substrate (the detail is shown in Figure S2). Ti_3_C_2_T_*x*_ MXene nanosheets were prepared by etching the Al layer. After blending MWCNT@MoS_2_ clusters and Ti_3_C_2_T_*x*_ MXene nanosheets, MXene/MWCNT@MoS_2_ heterogeneous films for EMI shielding were prepared by vacuum-assisted filtration.

**Figure 2:**
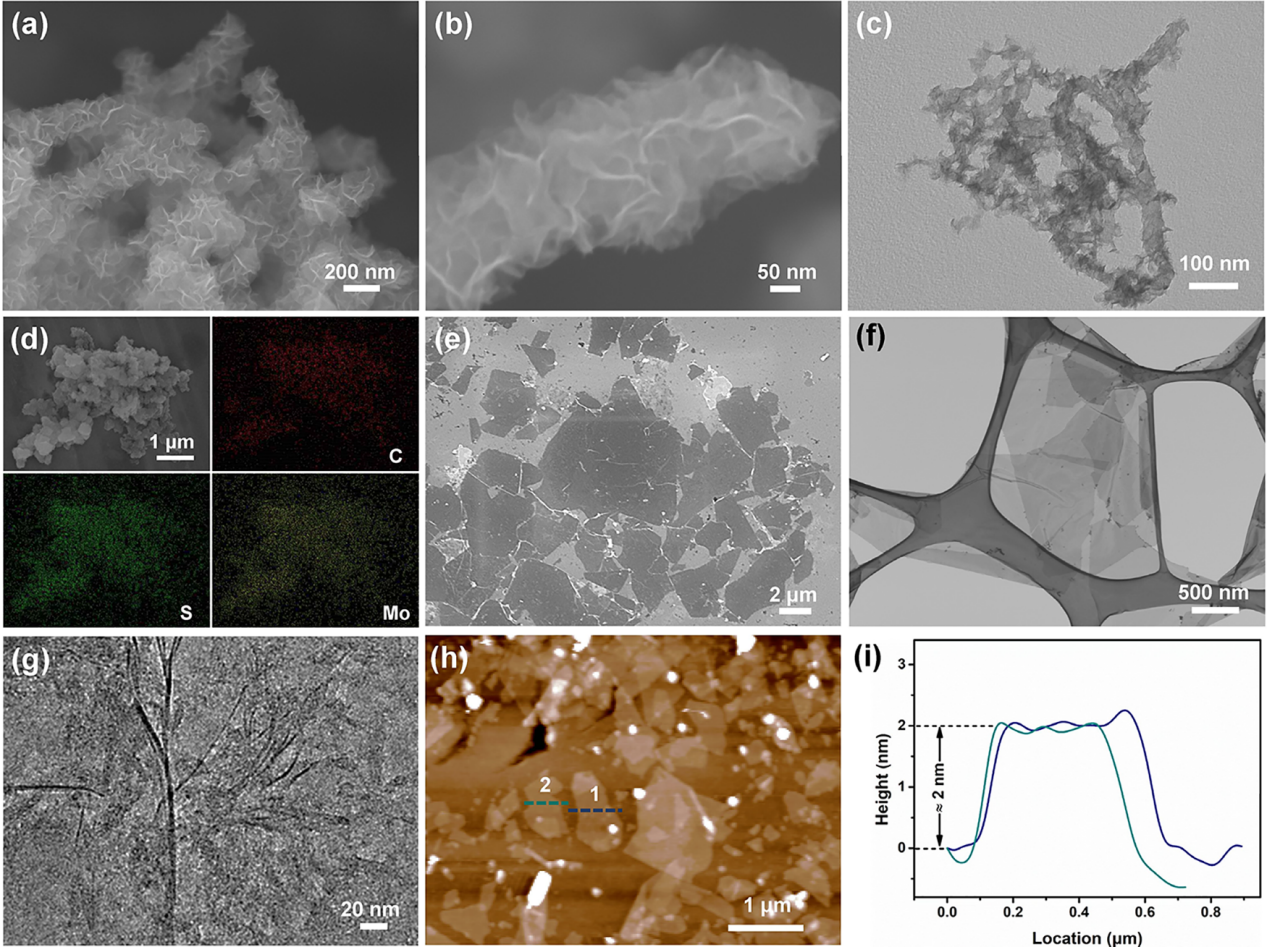
(a, b) FESEM images of the MWCNT@MoS_2_ cluster at different magnifications, (c) TEM image, and (d) elemental mappings of the MWCNT@MoS_2_ cluster. (e) SEM, (f) TEM, and (g) HRTEM images of Ti_3_C_2_T_*x*_ MXene nanosheets, (h) AFM image, and (i) height profile of Ti_3_C_2_T_*x*_ MXene nanosheets.

The as-synthesized MWCNT@MoS_2_ clusters were initially characterized by XRD (Figure S3). The characteristic peaks referring to the (002), (100), and (110) planes of MoS_2_ are found in the XRD pattern of MWCNT@MoS_2_, revealing the successful formation of MWCNT@MoS_2_ clusters. In addition, the peak at 26° can be ascribed to the (002) plane of MWCNTs. The morphology of as-synthesized MWCNT@MoS_2_ is shown in Figure 2a,b. The SEM images show that the MWCNT@MoS_2_ cluster is assembled by a one-dimensional (1D) structure with a diameter of about 250 nm (Figure 2a). Notably, this dimension is considerably larger in comparison to that of the functionalized MWCNT substrate (Figure S4). These MWCNT@MoS_2_ clusters are interconnected by MoS_2_ nanosheets that proliferate across the surface of the MWCNTs, forming a “hairy” structure. Upon closer inspection in Figure 2b, it becomes evident that the MoS_2_ nanosheets are densely packed onto the MWCNTs with a size of approximately 60–100 nm. In the synthesis system, thiourea served as both the sulfur source for MoS_2_ nanosheets and the linker between MoS_2_ nanosheets and acid-treated MWCNTs.^21^ This heterostructure was further validated by TEM images, which manifested the uniform growth of MoS_2_ nanosheets on the external surface of the MWCNTs (Figure 2c). The elemental mappings of MWCNT@MoS_2_ clusters (Figure 2d) corroborate the even distribution of MoS_2_ nanoflakes on the MWCNTs, in accordance with the SEM and TEM results. The energy-dispersive spectrometry analysis of MWCNT@MoS_2_ clusters (Figure S5) suggests an atomic ratio of 1:1.92 for Mo and S elements, providing further substantiation for the presence of MoS_2_ on the MWCNTs.

The SEM images of Ti_3_C_2_T_*x*_ MXene are shown in Figure 2e, in which the Ti_3_C_2_T_*x*_ MXene nanosheets are dispersed on a silicon wafer. It is found that the Ti_3_C_2_T_*x*_ MXene exhibits a well-exfoliated ultrathin nanosheet structure with lateral dimensions ranging from a few hundred nanometers to a few micrometers in the lateral dimension. Figure 2f shows a TEM image of Ti_3_C_2_T_*x*_ MXene nanosheets supported on lacey carbon. The transparency of the nanosheets with respect to the electron beam illumination verifies the ultrathin nature of the Ti_3_C_2_T_*x*_ MXene. Further evidence of good exfoliation is provided by the high-resolution TEM (HRTEM) image in Figure 2g, which reveals the presence of single layers and a few layers of Ti_3_C_2_T_*x*_ MXene nanosheets. The thickness of the Ti_3_C_2_T_*x*_ MXene nanosheets was measured by atomic force microscopy (AFM). The AFM image (Figure 2h,i) of Ti_3_C_2_T_*x*_ MXene nanosheets reveals an average thickness of 2.0 nm, confirming the ultrathin single-layered structure of the Ti_3_C_2_T_*x*_ nanosheet. Results from XRD analyses (Figure S6) demonstrate that distinctive peaks referring to the (101), (104), and (105) planes of Ti_3_AlC_2_ vanish after the etching process. Additionally, the (002) peak in the XRD pattern of Ti_3_C_2_T_*x*_ is shifted to a lower angle, signifying an increased interlayer distance within the Ti_3_C_2_T_*x*_ MXene structure.^24^

**Figure 3:**
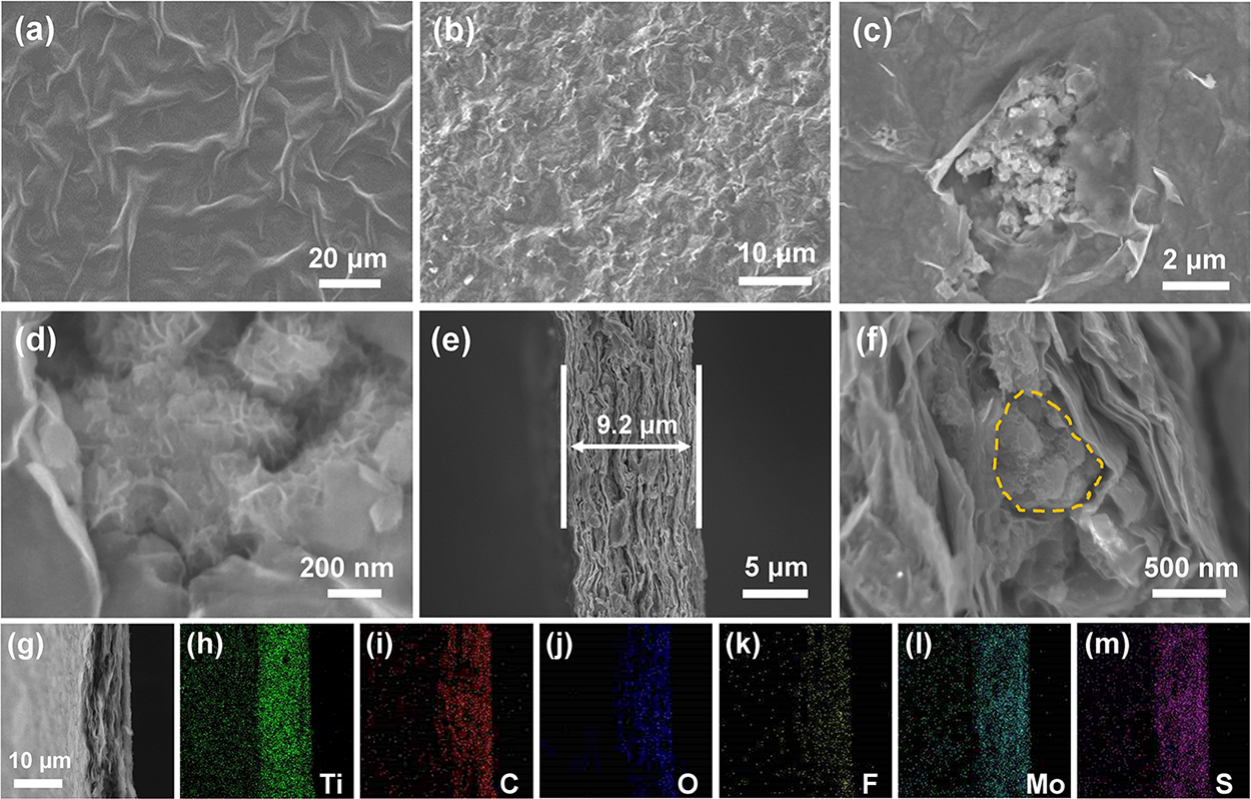
(a) Top-view image of Ti_3_C_2_T_*x*_ MXene film, (b–d) top-view images of MXene/MWCNT@MoS_2_-15%, (e) cross-section of Ti_3_C_2_T_*x*_ MXene, (f) cross-section of MXene/MWCNT@MoS_2_-15%, and (g–m) elemental mapping of the MXene/MWCNT@MoS_2_-15% film: (h) Ti, (i) C, (j) O, (k) F, (l) Mo, and (m) S.

The SEM images of both Ti_3_C_2_T_*x*_ MXene and MXene/MWCNT@MoS_2_-15% are shown in Figure 3a–f. Upon close examination, it becomes apparent that both pure Ti_3_C_2_T_*x*_ MXene and MXene/MWCNT@MoS_2_-15% heterogeneous films exhibit analogous surface characteristics characterized by prominent wrinkles (Figure 3a,b). However, some particles (irregular shape) appear on the surface of the heterogeneous film (Figure 3c,d), setting it apart from pure Ti_3_C_2_T_*x*_ MXene. The high-magnification SEM image (Figure 3d) provides an enhanced view, clearly illustrating the embedding of MWCNT@MoS_2_ clusters in Ti_3_C_2_T_*x*_ MXene layers, ultimately forming a heterogeneous structure. The cross-sectional SEM image of MXene/MWCNT@MoS_2_-15% showcases a well-aligned, layered structure with an approximate thickness of 9.2 μm (Figure 3e). Moreover, the high-magnification cross-sectional SEM image (Figure 3f) underscores the presence of MWCNT@MoS_2_ nanoclusters (indicated by orange dashed lines) embedded within the Ti_3_C_2_T_*x*_ MXene layers, consistent observation with the top-view observations of MXene/MWCNT@MoS_2_-15%. This structural configuration, wherein MWCNT@MoS_2_ clusters are integrated into the Ti_3_C_2_T_*x*_ MXene layers, leads to an expanded interlayer spacing of the Ti_3_C_2_T_*x*_ MXene nanosheet, thereby affording additional porous structures within the hybrid films. The elemental mapping of MXene/MWCNT@MoS_2_-15% (Figure 3g–m) presents an even distribution of Ti, C, O, F, Mo, and S throughout the heterogeneous film, indicative of the successful blending of Ti_3_C_2_T_*x*_ nanosheets and MWCNT@MoS_2_ clusters.

**Figure 4:**
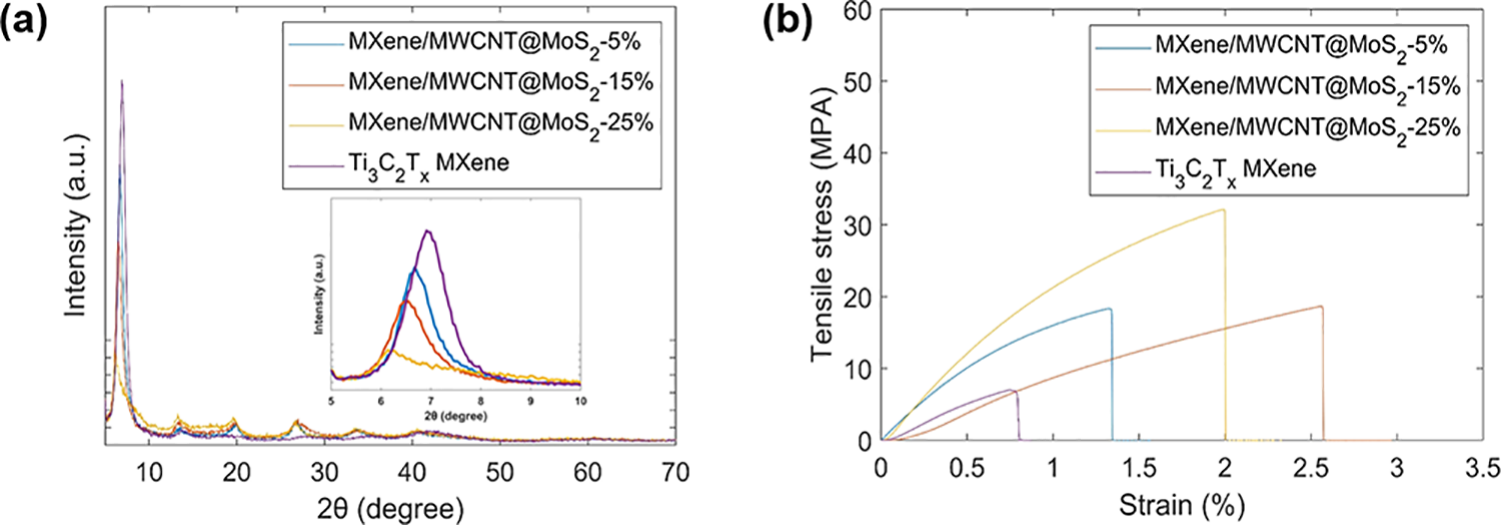
(a) XRD pattern, inset: enlarged (002) diffraction peaks and (b) tensile stress–strain curves of Ti_3_C_2_T_*x*_ MXene and MXene/MWCNT@MoS_2_ heterogeneous films.

The thin films were examined by using XRD to assess alterations in the interplanar distance between the MXene nanosheets. Figure 4a offers a comparison of the XRD patterns between the MXene/MWCNT@MoS_2_ heterogeneous film and pure Ti_3_C_2_T_*x*_. The XRD pattern of pure MXene exhibits a prominent (002) diffraction peak at 6.9°, corresponding to an interlayer spacing of 1.28 nm, which is the characteristic of Ti_3_C_2_T_*x*_ MXene with water molecules located between its layers.^25^ This 2θ value closely aligns with that reported in the literature for pure MXene.^25^ Upon introduction of MWCNT@MoS_2_ clusters, the (002) diffraction peak shifts to lower angles and experiences a reduction in intensity, suggesting the presence of MWCNT@MoS_2_ clusters between MXene layers. Furthermore, with an increase in the MWCNT@MoS_2_ content, the intensity of the (002) peak diminishes, indicating a reduction in the extent of stacking order within MXene layers. The tensile testing results (Figure 4b) underscore the exceptional mechanical resilience exhibited by the heterogeneous films following the incorporation of MWCNT@MoS_2_. Compared with the pure Ti_3_C_2_T_*x*_ MXene film, the heterogeneous films featuring MWCNT@MoS_2_ demonstrate significant improvements in mechanical properties, including enhanced tensile strength, toughness, and failure strain. The tensile strength of the pure Ti_3_C_2_T_*x*_ MXene film stands at 5.3 MPa, accompanied by a failure strain of 0.8%, similar to values reported in ref (26). By increasing the quantity of MWCNT@MoS_2_ by 1, 3, and 5 mg, the tensile strength experiences substantial increments, reaching approximately 16, 17, and 32 MPa, respectively. Correspondingly, the fracture strains are measured at 1.36, 2.57, and 2.02%, respectively. Remarkably, the MXene/MWCNT@MoS_2_-15% heterogeneous film exhibits the highest failure strain and a significantly enhanced tensile strength in comparison to the pure Ti_3_C_2_T_*x*_ MXene film.

**Figure 5:**
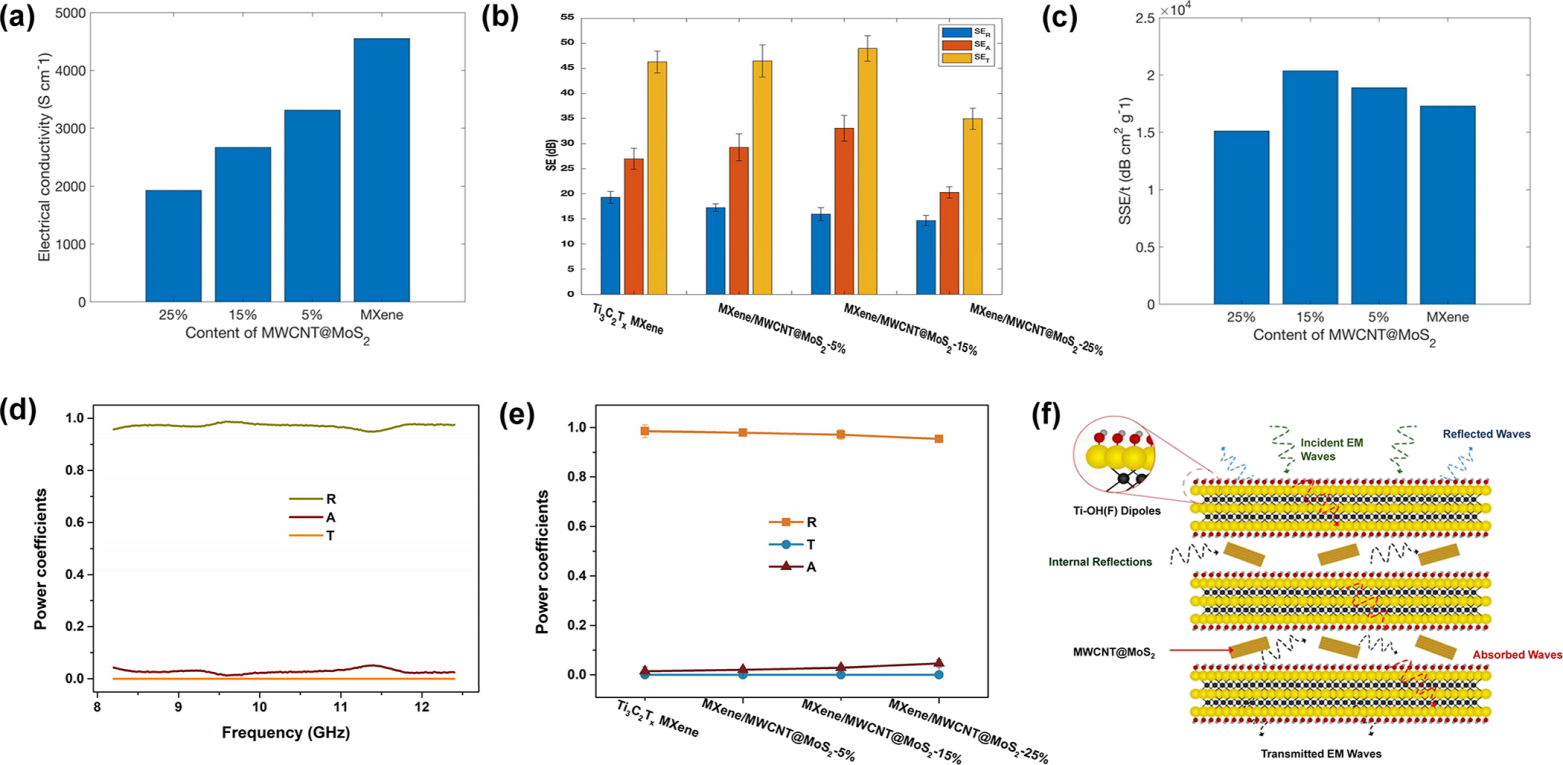
(a) Electrical conductivity of all studied samples. (b) Average SE_*A*_, SE_*R*_, and SE_T_ values for Ti_3_C_2_T_*x*_ MXene and heterogeneous films. (c) Comparison of EMI SSE/*t* of Ti_3_C_2_T_*x*_ and heterogeneous films. (d) Power coefficients (*T*, *A*, and *R*) of MXene/MWCNT@MoS_2_-15%. (e) Comparison of power coefficients (*T*, *A*, and *R*) of Ti_3_C_2_T_*x*_ MXene films and MXene/MWCNT@MoS_2_ heterogeneous films. (f) Proposed mechanism of EMI shielding for the heterogeneous film.

The electrochemical conductivity of the samples plays a crucial role in determining the EMI shielding performance. Among the samples examined, Ti_3_C_2_T_*x*_ films exhibited the highest electrical conductivity, reaching 4552 S cm ^–1^ (Figure 5a), aligning closely with the findings reported.^14^ As mentioned elsewhere,^27^ MXene with the surface termination displays conductivity due to the presence of free electrons in the surface termination, which actively contributes to the material’s electrical conductivity. As the content of MWCNT@MoS_2_ in the heterogeneous film increases, the electrical conductivity of the film experiences a reduction. This can be attributed to two factors: a decrease in the degree of stacking order of the MXene layers and the inclusion of MWCNT@MoS_2_ between the Ti_3_C_2_T_*x*_ nanosheets. The electrical conductivities for MXene/MWCNT@MoS_2_-5%, MXene/MWCNT@MoS_2_-15%, and MXene/MWCNT@MoS_2_-25% are 3312, 2671, and 1927 S cm ^–1^, respectively (Figure 5a). The complex permittivity (ε = ε′ + *i*ε″) of the as-synthesized samples has been investigated within the frequency range of 8.2–12.4 GHz. The real part (ε′) and the imaginary part (ε″) of the complex permittivity characterize the ability to produce polarization and the dissipation of energy, respectively.^6^ As shown in Figure S7a,b, MXene/MWCNT@MoS_2_-15% shows a high ε′ and the highest ε″, indicating its exceptional electrical conductivity and increased polarization capability.^28^Figure S7c illustrates the dielectric response (tangent loss) for the synthesized films. Intriguingly, the dielectric loss is most pronounced for the MXene/MWCNT@MoS_2_-15% film compared to other films, signifying that MXene/MWCNT@MoS_2_-15% possesses a more substantial dielectric loss capacity, including electrical conductivity, dipole relaxation, and interfacial polarization.

The results obtained from EMI shielding tests are shown in Figure 5b, with the total mass of the samples held constant. The total EMI SE (SE_T_), absorption SE (SE_*A*_), and reflection SE (SE_*R*_) of pure Ti_3_C_2_T_*x*_ MXene stand at 46, 27, and 19 dB, respectively, at a frequency range of f 8.2–12.4 GHz (X band). With an increase in the proportion of MWCNT@MoS_2_, both SE_T_ and SE_*A*_ of the heterogeneous films exhibit a continuous rise until they peak at 15% MWCNT@MoS_2_ and then decline at 25% MWCNT@MoS_2_. When the mass ratio of MWCNT@MoS_2_ to Ti_3_C_2_T_*x*_ MXene is 3:17 (MWCNT@MoS_2_-15% ), both SE_T_ and SE_*A*_ reach the highest values of 50 and 33 dB, respectively, suggesting the incorporation of MWCNT@MoS_2_ clusters into the film layers is beneficial to enhancing the adsorption of EMWs fueled by the induced porous structure and additional inner space of heterogeneous films.^16^ Conversely, the SE_*R*_ heterogeneous films exhibit a continuous decrease with the growing proportion of MWCNT@MoS_2_. This can be explained by the decline in the electrical conductivity of the heterogeneous film (Figure 5a). However, adding an excessive amount of MWCNT@MoS_2_ clusters can lead to poor shielding performance, which interpreted that the MWCNT@MoS_2_ cluster in Ti_3_C_2_T_*x*_ weakens the contact between the Ti_3_C_2_T_*x*_ layers, resulting in reduced electrical conductivity and, subsequently, diminished shielding performance. Although pure Ti_3_C_2_T_*x*_ films do not have substantial pores compared to MXene/MWCNT@MoS_2_ heterogeneous films, their extraordinarily high electrical conductivity sustains their high SE_T_. In summary, the introduced MWCNT@MoS_2_ can adjust the spacing between Ti_3_C_2_T_*x*_ layers and suppress the stacking of Ti_3_C_2_T_*x*_ nanosheets, thus further optimizing the EMI shielding performances of Ti_3_C_2_T_*x*_-based films.

The relative densities of Ti_3_C_2_T_*x*_ MXene, MXene/MWCNT@MoS_2_-5%, MXene/MWCNT@MoS_2_-15%, and MXene/MWCNT@MoS_2_-25% are calculated to be 2.23, 1.64, 1.56, and 1.36 g cm ^–3^, respectively. This decreasing density trend is due to the lower density of MWCNT@MoS_2_ clusters (0.21 g cm ^–3^), which results in a reduction in the overall density of the MXene/MWCNT@MoS_2_ heterogeneous films as the MWCNT@MoS_2_ content increases. The thicknesses of Ti_3_C_2_T_*x*_ MXene, MXene/MWCNT@MoS_2_-5%, MXene/MWCNT@MoS_2_-15%, and MXene/MWCNT@MoS_2_-25% are measured to be 12, 15, 16, and 17 μm, respectively. Finally, the absolute effectiveness (SEE/*t*) (Figure 5c) is calculated to be 17,292, 18,891, 20,355, and 15,110 for Ti_3_C_2_T_*x*_ MXene, MXene/MWCNT@MoS_2_-5%, MXene/MWCNT@MoS_2_-15%, and MXene/MWCNT@MoS_2_-25%, respectively. Notably, although SE_*R*_ and SE_*A*_ provide valuable insights into the EMI shielding performance, they do not individually signify the proportion of EMW reflection and absorption.^29,30^ In order to delve deeper into the EMI shielding mechanism of MXene/MWCNT@MoS_2_ heterogeneous films, an examination of the absorption (*A*), reflection (*R*), and transmission (*T*) coefficients is pursued. The coefficients of MXene/MWCNT@MoS_2_-15% are illustrated in Figure 5d. The near-zero values of *T* indicate that the films allow only a negligible portion of EMWs to traverse through them. This observation strongly implies the exceptional EMI shielding efficacy exhibited by the films. Moreover, upon contrasting the absorption (*A*) values, it becomes evident that significantly higher reflection (*R*) values (>0.9) are attained. This outcome underscores that the mechanism primarily responsible for EMW shielding in MXene/MWCNT@MoS_2_ heterogeneous films is the EMW reflection. It is worth noting that the absorption (*A*) values exhibit a subtle rise with the increasing content of MWCNT@MoS_2_ within the heterogeneous films (Figure 5e). This observation highlights that the incorporation of MWCNT@MoS_2_ contributes to the enhancement of EMW absorption despite the concurrent reduction in electrical conductivity.

Further, the progressive increase in EMI SE as the film thickness increases (Figure S8) is a result of the longer path length that EMW must traverse within the MXene/MWCNT@MoS_2_ films. As the film gets thicker, EMW encounters more material, leading to greater energy loss during its passage through the film. To aid in understanding the EMI shielding mechanism of the heterogeneous film, a schematic representation is provided in Figure 5f. Some EMWs are directly reflected as they strike the surface of the hybrid film due to the abundant free electron of the conductive MXene/MWCNT@MoS_2_ heterogeneous film, leading to huge conductive loss of the EMWs. The remaining waves penetrate via the MXene lattice structure, where interaction with the surface of high electron density causes currents that contribute to ohmic losses, leading the EMWs to lose their energy. The multiple heterogeneous interfaces between MXene and embedded MWCNT@MoS_2_ clusters can result in the accumulation and inhomogeneous distribution of charge carriers, leading to intensive interfacial polarization and the associated relaxation.^31,32^ In alternative EM fields, the unevenly distributed charges caused by the interface polarization, are compelled to orderly rearrange following the propagation direction of the EM field, leading to the dissipation of EM energy in the dynamic equilibrium of the polarization and relaxation processes.^33^ The porous structure induced by MWCNT@MoS_2_ clusters inserting MXene layers can provide extra space for multiple internal reflections of EMWs to further adsorb more EMWs. Besides, the numerous MoS_2_ nanosheets provide abundant polarized sites and void space, further improving the EM attenuation ability.^32^

**Figure 6:**
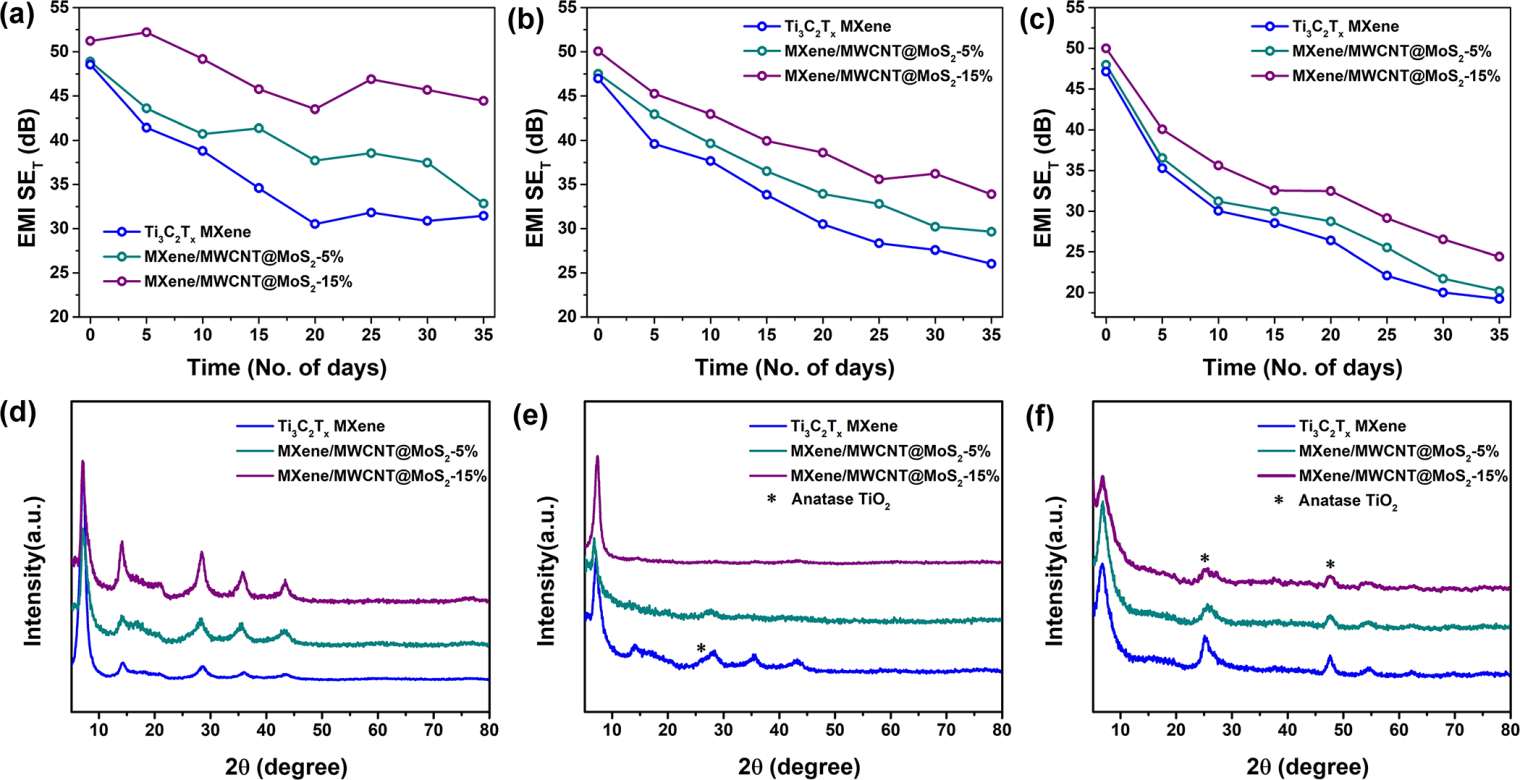
Total EMI SE vs time measurements and XRD patterns for Ti_3_C_2_T_*x*_ MXene, MXene/MWCNT@MoS_2_-5%, and MXene/MWCNT@MoS_2_-15%. (a, d) in a desiccator, (b, e) under ambient laboratory conditions (55% RH), and (c, f) in an environmental chamber (90% RH). Connecting lines in parts a–c are for indication of data trend only.

The Ti_3_C_2_T_*x*_ MXene, MXene/MWCNT@MoS_2_-5%, and MXene/MWCNT@MoS_2_-15% films were chosen for long-term stability tests under different storage conditions. These films were stored under three different conditions: a desiccator, ambient laboratory conditions, and an environmental chamber at 90% relative humidity (RH). As seen in Figure 6a, the EMI SE_T_ of all three samples experienced a decrease in their SE_T_, when preserved in a desiccator for 35 days. The Ti_3_C_2_T_*x*_ MXene film showed a ∼35% reduction in SE_T_, while MXene/MWCNT@MoS_2_-15% experienced a milder drop of 13%. Storage under ambient laboratory conditions with ∼55% RH caused a significant decline in the EMI shielding performance for all samples (Figure 6b). This reduction is attributed to the hydrophilic nature of Ti_3_C_2_T_*x*_ MXene, which contains functional groups such as −OH, −F, and −O–. When exposed to moisture, Ti_3_C_2_T_*x*_ can oxidize, forming nonconductive TiO_2_, thus reducing electrical conductivity.^34^ After 35 days under these conditions, SE_T_ dropped by ∼32% for MXene/MWCNT@MoS_2_-15%, which is lower than the reduction observed for MXene/MWCNT@MoS_2_-5% (∼38% ) and Ti_3_C_2_T_*x*_ MXene (∼45% ). These results suggest that MXene/MWCNT@MoS_2_-15% exhibits greater stability under ambient laboratory conditions when compared to MXene/MWCNT@MoS_2_-5% and Ti_3_C_2_T_*x*_ MXene. Films stored in an environmental chamber at 90% RH experienced the most significant degradation in the EMI shielding performance, as shown in Figure 6c. This environment led to violent oxidation of Ti_3_C_2_T_*x*_ MXene,^35^ causing a substantial decrease in shielding effectiveness for all films. Despite all of the films experiencing a significant decrease in performance in an environmental chamber at 90% RH, MXene/MWCNT@MoS_2_-15% demonstrates the highest retention of performance compared to the other two films. In summary, under all three conditions, MXene/MWCNT@MoS_2_-15% exhibits the highest level of performance retention when compared to the other two films. This highlights the critical role of integrating materials that can shield MXene-based EMI from moisture exposure.

After the stability test, the films can still exhibit high SE_T_ values of above 30 dB in a desiccator and ambient condition, which is significantly higher than those observed in the environmental chamber (less than 25 dB). This difference in stability can be attributed to the varying moisture levels in these environments, which can lead to different degrees of oxidization for MXene-based films. The response of the films to different environmental conditions is further elucidated by XRD patterns, as shown in Figure 6d–f. In a desiccator and ambient laboratory conditions (55% RH), almost all three films can maintain their original MXene phase. However, in the highly humid environmental chamber (90% RH), the XRD patterns of all three films reveal the presence of peaks of (101) and (200) planes associated with anatase TiO_2_,^36−38^ indicating the poor structural stability of the samples under high RH. Interestingly, MXene/MWCNT@MoS_2_-15% shows the lowest peak density for anatase TiO_2_ in the environmental chamber, confirming its superior stability compared to the other two samples. The improved structural stability of MXene/MWCNT@MoS_2_-15% can be attributed, in part, to its enhanced hydrophobic properties, as is evident from the wetting behaviors of the films (Figure S9). The Ti_3_C_2_T_*x*_ MXene film displays a hydrophilic nature with a low water contact angle of 44.7°. However, after the introduction of MWCNT@MoS_2_ clusters, the composite films exhibit higher contact angles and reach the maximum value of 79.7° for MXene/MWCNT@MoS_2_-25%. The MXene/MWCNT@MoS_2_-15% with a water contact angle of 57.0° shows a more hydrophobic nature compared to pure Ti_3_C_2_T_*x*_ MXene. Moreover, the surface microstructure of the film has a significant effect on the structural stability. The rough surface of MXene/MWCNT@MoS_2_ is able to induce hydrophobic properties, a phenomenon well-documented in previous scientific studies.^39−41^ Furthermore, the replacement of some Ti_3_C_2_T_*x*_ MXene with MWCNT@MoS_2_ in the film reduced the exposure of vulnerable Ti_3_C_2_T_*x*_ MXene flakes, thereby diminishing the detrimental oxidation process. This combination of hydrophobicity and altered surface microstructure likely contributed to the improved stability of MXene/MWCNT@MoS_2_-15% under environmental conditions.

**Figure 7:**
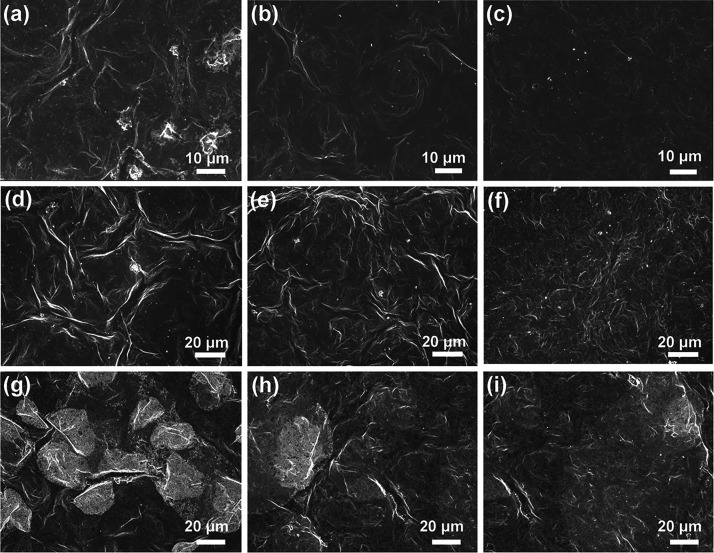
Surface SEM images of (a, d, g) Ti_3_C_2_T_*x*_ MXene, (b, e, h) MXene/MWCNT@MoS_2_-5%, and (c, f, i) MXene/MWCNT@MoS_2_-15% under different environmental conditions: a desiccator (a–c), ambient laboratory condition (55% RH) (d–f), and an environmental chamber (90% RH) (g–i).

Figure 7 illustrates the surface morphology of Ti_3_C_2_T_*x*_ MXene, MXene/MWCNT@MoS_2_-5%, and MXene/MWCNT@MoS_2_-15% under various environmental conditions: a desiccator, ambient laboratory conditions (55% RH), and an environmental chamber (90% RH). The images clearly illustrate the changes in surface appearance as humidity levels increase. Under all three conditions, Ti_3_C_2_T_*x*_ MXene experiences severe oxidation, as evidenced by the presence of numerous TiO_2_ nanoparticles on the film’s surface (the most severe oxidization happened in an environmental chamber (90% RH)), as shown in Figure 7a,d,g. Comparatively, MXene/MWCNT@MoS_2_-5% shows better stability than pure MXene, with fewer TiO_2_ particles observed on the surface under both dry and humid conditions (Figure 7b,e,h). Remarkably, MXene/MWCNT@MoS_2_-15% exhibits a significantly higher degree of stability, as only a few TiO_2_ particles are sparsely distributed on its surface under both dry and humid conditions (Figure 7c,f,i).

## 4 Conclusions

In this study, we successfully synthesized flexible, lightweight, and ultrathin MXene/MWCNT@MoS_2_ heterogeneous films with exceptional electrical conductivity and demonstrated their enhanced EMI shielding performance. The introduction of MWCNT@MoS_2_ clusters provides additional active sites and space and effectively prevents the restacking of Ti_3_C_2_T_*x*_ MXene flakes. Therefore, the MXene/MWCNT@MoS_2_ heterogeneous film exhibited higher SE_T_ and SSE/*t* values compared to pure MXene films. Furthermore, the mechanical properties and environmental stability of the heterogeneous film were significantly enhanced, making it a promising material for various applications, including smart and wearable electronic devices. However, it is important to note that the EMI shielding performance of the heterogeneous film showed some degradation in moisture environments, despite the enhancement provided by the MWCNT@MoS_2_ clusters. Further research is needed to develop more effective strategies to enhance the environmental stability of these films, especially under high humidity conditions.

## Supporting Information Available

Material characterization, synthesis illustration, SEM images, XRD patterns, EDX, contact angle, EMI shielding measurement (PDF)
