# Supplementary material for: Heterogeneous Ti3C2Tx MXene-MWCNT@MoS2 Film for Enhanced Long-Term Electromagnetic Interference Shielding in the Moisture Environment
Source: ACS Appl Mater Interfaces. 2023 Oct 16;15(42):49458–67. doi: 10.1021/acsami.3c08279 (PMC10614194; doi:10.1021/acsami.3c08279)
Supplement: Supplementary file 1 — am3c08279_si_001.pdf [file am3c08279_si_001.pdf]

# Supporting Information

## Heterogeneous $Ti_3C_2T_x$ MXene-MWCNT@$MoS_2$ film for enhanced long-term electromagnetic interference shielding in moisture environment

*Sarab Ahmed [a,&], Baosong Li [b,&], Shaohong Luo [c], Kin Liao [a, c, *]*

[a] Department of Aerospace Engineering, Khalifa University of Science and Technology, 127788, Abu Dhabi, UAE

[b] Department of Chemical Engineering, Khalifa University of Science and Technology, 127788, Abu Dhabi, UAE

[c] Department of Mechanical Engineering, Khalifa University of Science and Technology, 127788, Abu Dhabi, UAE

[&] Equal contribution

[*] **Corresponding author**: Kin Liao, kin.liao@ku.ac.ae

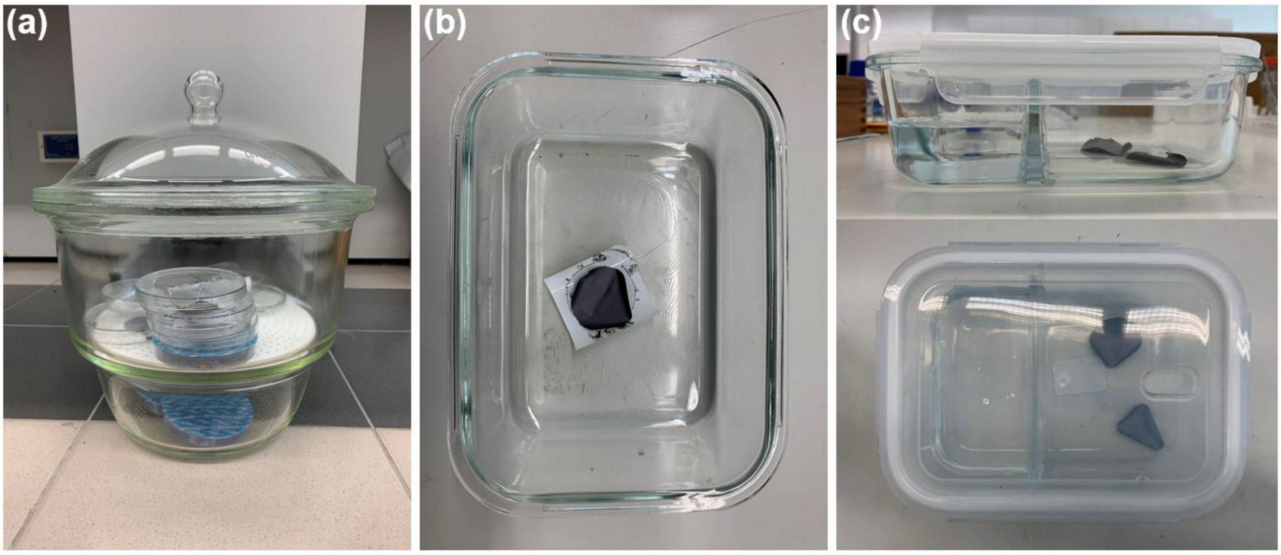


**Figure S1** Three type of containers used in the environmental experiment: (a) a desiccator with desiccant ( < 10% RH), (b) a container in open air (55% RH), (c) an environmental chamber made of a sealed small container with water inside (90% RH).

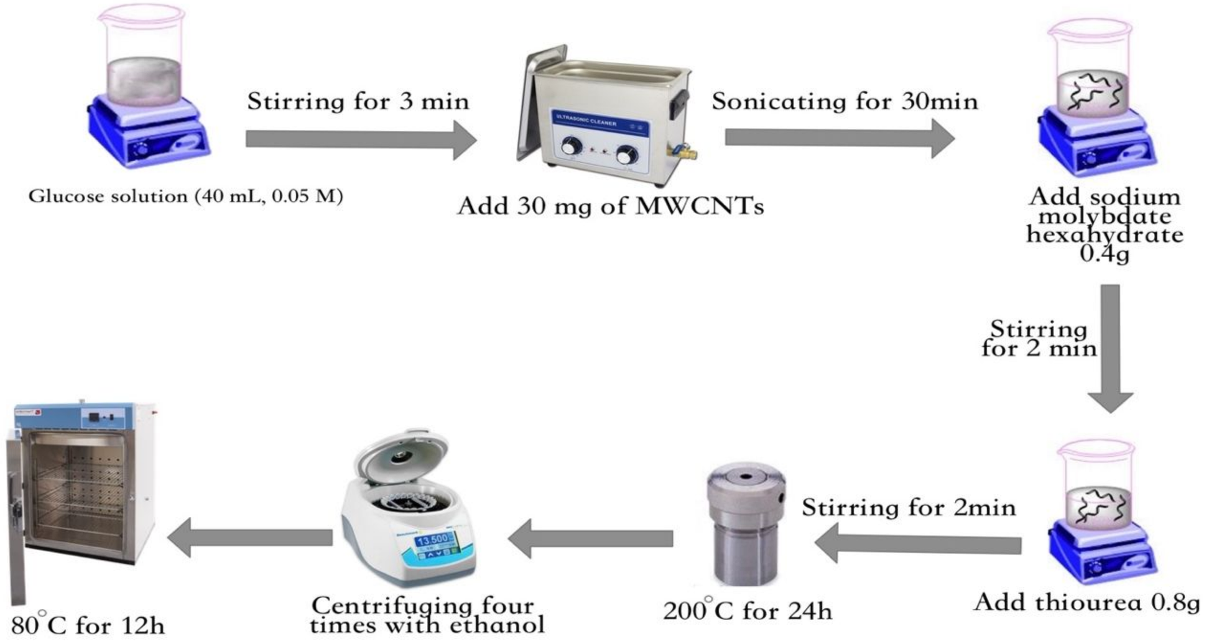


**Figure S2** The entire experimental process of preparing MWCNT@$MoS_2$.

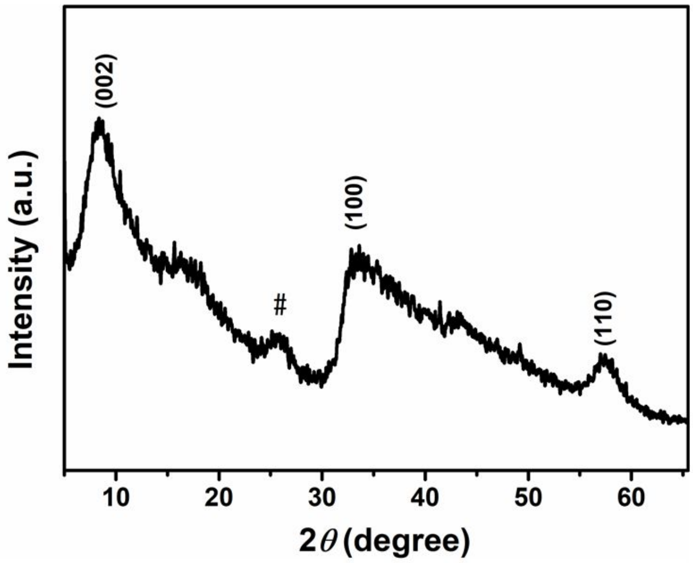


**Figure S3** XRD pattern of as-prepared MWCNT@$MoS_2$.

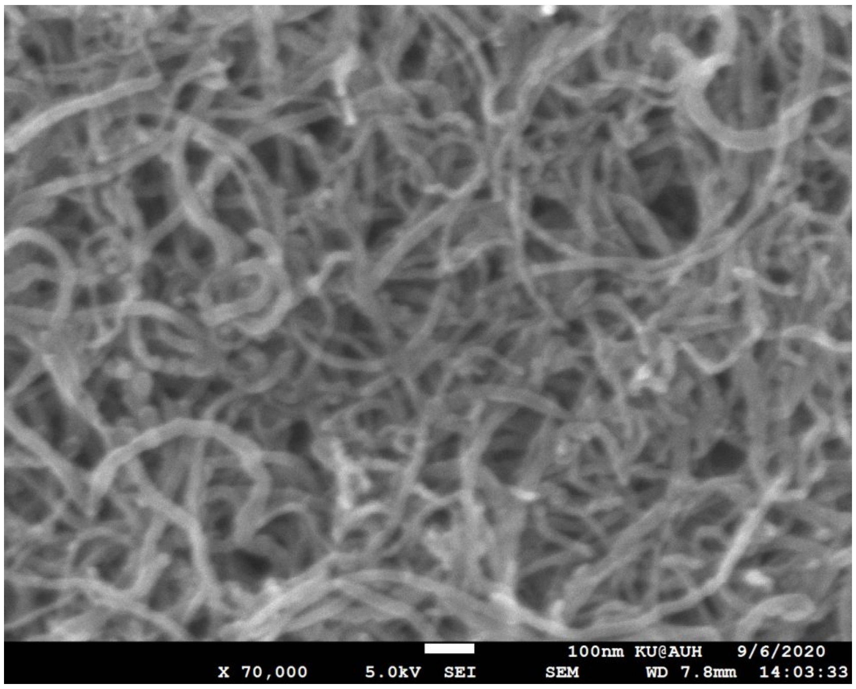

**Figure S4** SEM image of acid-treated MWCNT.

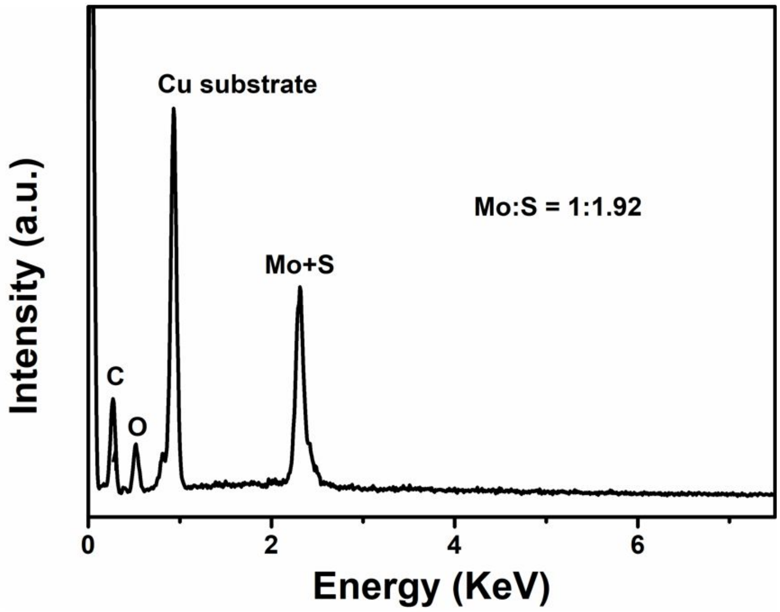


**Figure S5** EDS of as-prepared MWCNT@$MoS_2$.

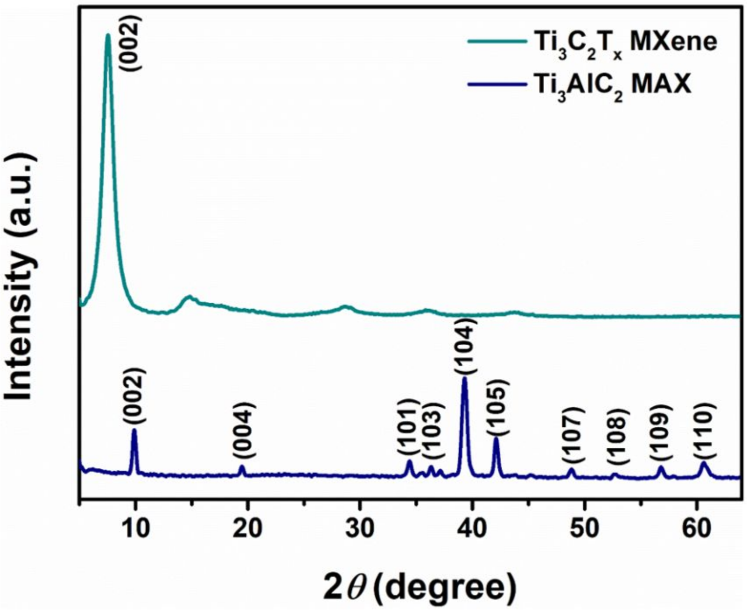


**Figure S6** XRD pattern of $Ti_3AlC_2$ MAX and $Ti_3C_2T_x$ MXene.

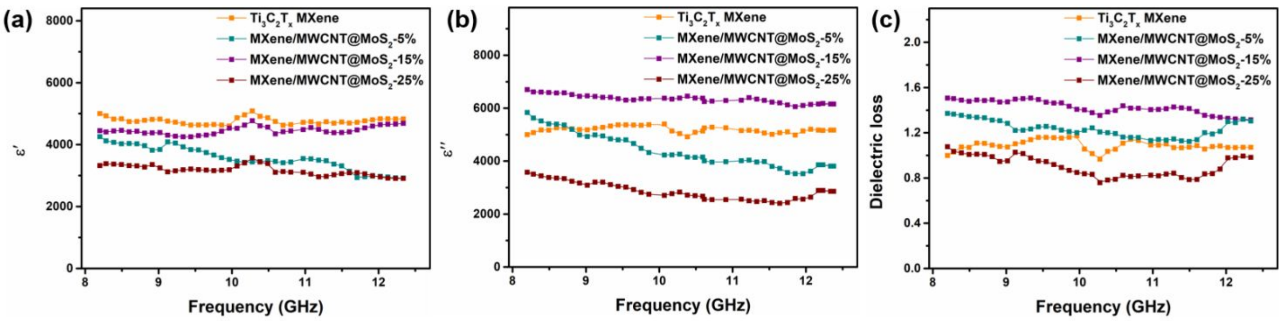


**Figure S7** (a) Real permittivity, (b) imaginary permittivity, (c) dielectric loss of the as-synthesized sample in the X-band frequency range.

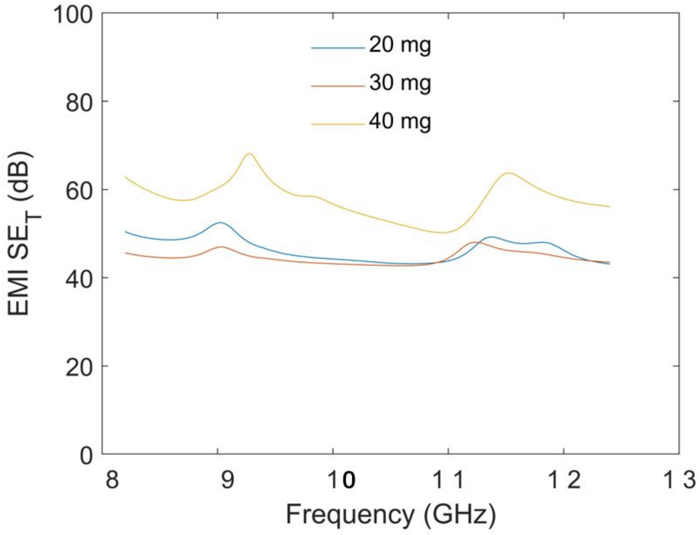


**Figure S8** (a) EMI $SE_T$ of MXene/MWCNT@$MoS_2$-15% with different masses.

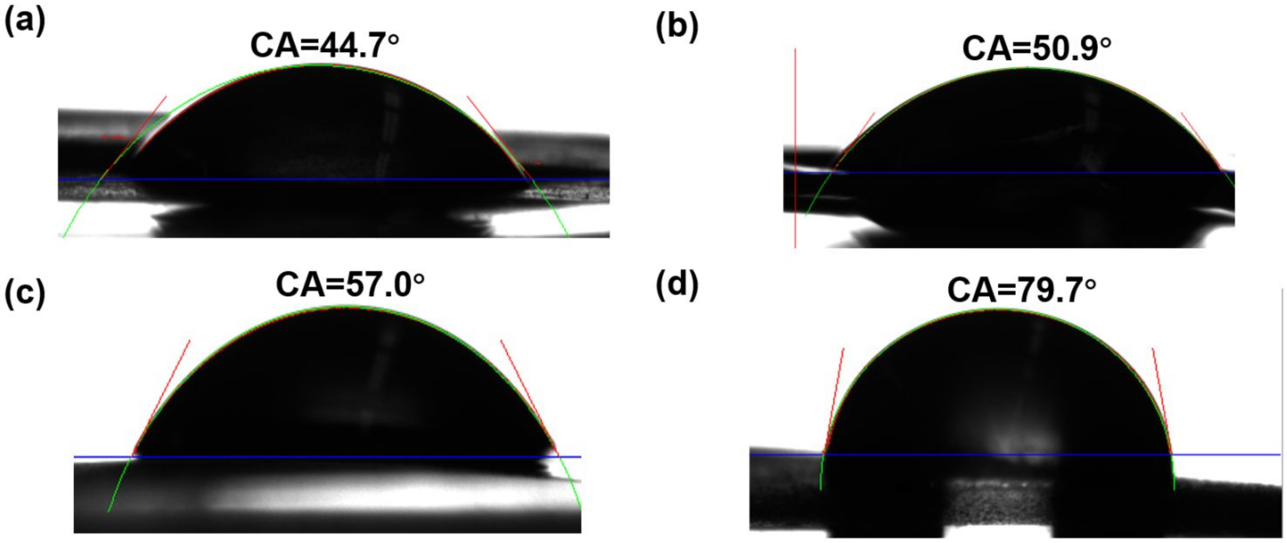


**Figure S9** Contact angles of (a) $Ti_3C_2T_x$ MXene and (b-c) MXene/MWCNT@$MoS_2$ heterogeneous films: (b) 5%, (c) 15%, and (d) 25%.
